# Life satisfaction and self-rated health in long-term survivors after extracorporeal cardiopulmonary resuscitation (ECPR): associations with functional outcome, cognition, anxiety, depression, fatigue, and physical activity

**DOI:** 10.1016/j.resplu.2026.101521

**Published:** 2026-10-07

**Authors:** Anna Henningsson, Pia Watson, Magnus Segerström, Petra Redfors, Bengt Redfors

**Affiliations:** a Department of Cardiothoracic Anesthesia and Intensive Care, Sahlgrenska University Hospital, Gothenburg, Region Västra Götaland, Sweden; b Department of Anesthesiology and Intensive Care Medicine, Sahlgrenska Academy, University of Gothenburg, Gothenburg, Sweden; c Department of Cardiology, Sahlgrenska University Hospital, Gothenburg, Region Västra Götaland, Blå Stråket 5, 413 45 Gothenburg, Sweden; d Department of Neurology, Sahlgrenska University Hospital, Gothenburg, Region Västra Götaland, Sweden; e Department of Clinical Neuroscience, Institute of Neuroscience and Physiology, Sahlgrenska Academy, University of Gothenburg, Gothenburg, Sweden

**Keywords:** Cardiac arrest, Cardiopulmonary resuscitation, Extracorporeal membrane oxygenation, Health-related quality of life, Patient reported outcome measures, Life satisfaction

## Abstract

**Background:**

The goal of extracorporeal cardiopulmonary resuscitation (ECPR) is not only survival, but long-term satisfaction with life. Whether functional outcome (modified Rankin Scale; mRS), cognition, emotional symptoms, fatigue, and physical activity relate to life satisfaction in ECPR survivors remain unknown. We evaluated these aspects of health and their association with life satisfaction and self-rated health.

**Methods:**

This cross-sectional follow-up included survivors ≥1 year after ECPR at Sahlgrenska University Hospital, Gothenburg, Sweden (2010–2021). Primary outcomes were life satisfaction (World Values Survey) and self-rated health (EQ-5D-5L VAS). Secondary outcomes were mRS, Montreal Cognitive Assessment (MoCA), Hospital Anxiety and Depression Scale (HADS), Mental Fatigue Scale (MFS), and self-reported physical activity. Exploratory multivariable linear regression assessed associations with primary outcomes.

**Results:**

Of 34 survivors, 26 (76.5%) participated; median follow-up was 5 [IQR 2–7] years; 96% had mRS 0–3. Median life satisfaction was 8 [6–9]/10 and self-rated health 81 [70–90]/100, similar to population reference values (7.55 and 80). Positive screen for cognitive impairment (MoCA < 26) was present in 56%, anxiety (HADS-A ≥ 8) 42%, depression (HADS-D ≥ 8) 15%, and fatigue (MFS ≥ 10.5) 50%. In exploratory multivariable analysis, moderate physical activity was associated with life satisfaction (*B* 0.36 per day/week; 95% CI 0.06–0.67; *p* = 0.023) and self-rated health (*B* 4.60; 0.71–8.50; *p* = 0.024).

**Conclusion:**

Long-term ECPR survivors reported life satisfaction and self-rated health similar to population reference values. In exploratory multivariable analysis, moderate physical activity was the only variable associated with both. This supports assessing life satisfaction and physical activity in post-ECPR follow-up.

## Highlights

• First study to assess life satisfaction in long-term ECPR survivors.
• Life satisfaction and self-rated health were similar to population reference values.
• Positive screens for cognitive impairment, anxiety and fatigue were common.
• Physical activity was associated with both life satisfaction and self-rated health.
• Whether physical activity can improve well-being after ECPR remains to be tested.

## Graphical abstract

**Figure:**
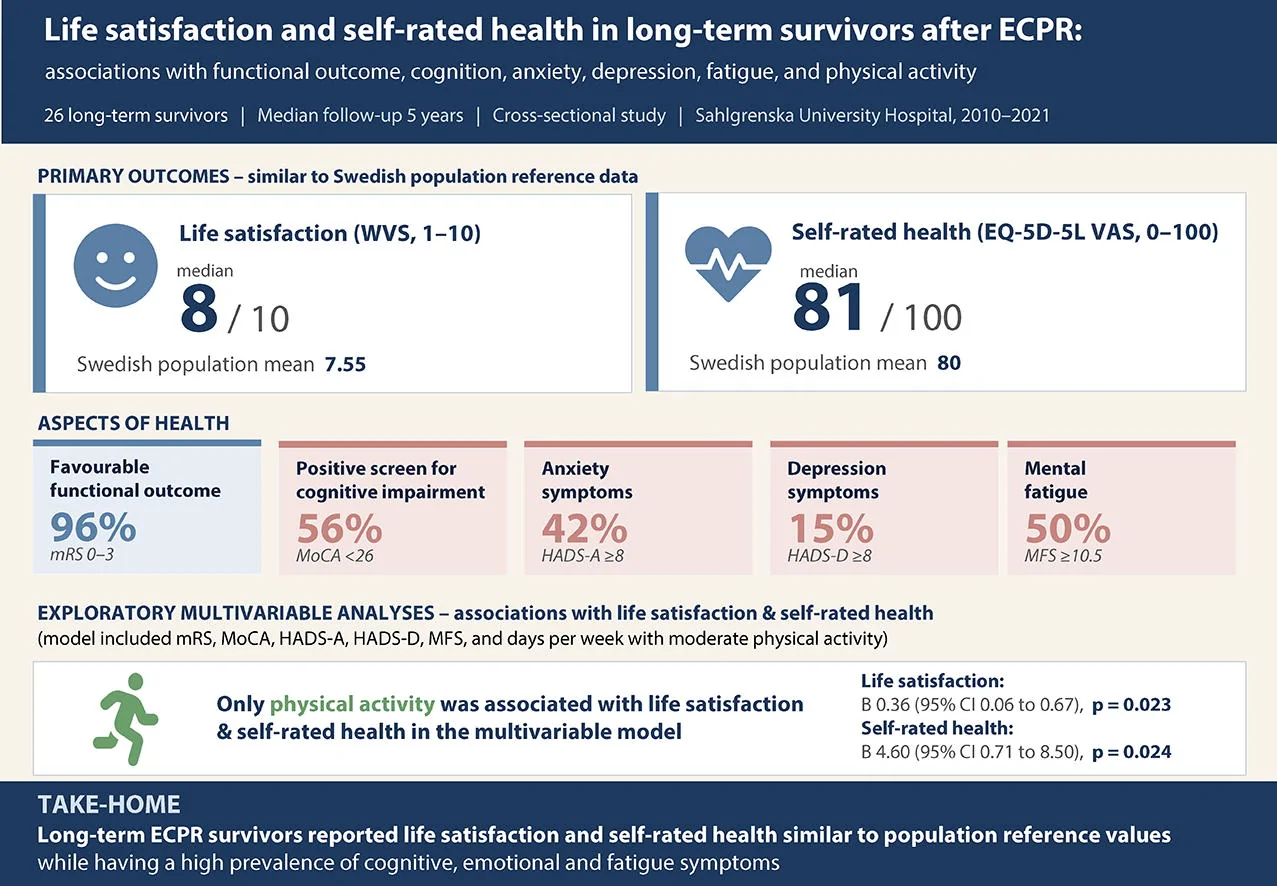

## Introduction

For selected patients with refractory cardiac arrest (CA), extracorporeal cardiopulmonary resuscitation (ECPR) can restore circulation when conventional resuscitation fails. Current guidelines recommend ECPR as a rescue therapy in selected patients, but it is costly, requires specialised teams, is initiated under substantial prognostic uncertainty, and often involves prolonged intensive care that competes with other patients for limited capacity.^1^, ^2^ As ECPR programmes expand, centres considering implementation therefore need data not only on survival and functional outcome, but also on whether survivors regain a life they themselves consider meaningful.^3^

Outcome after CA is commonly reported as survival or favourable functional outcome, often defined as a modified Rankin Scale (mRS) score of 0–3.^4^, ^5^, ^6^ These measures are essential, but they may not fully capture survivors’ long-term health or lived experience.^5^, ^6^, ^7^, ^8^ European Resuscitation Council (ERC) post-resuscitation guidelines recommend assessment of cognition, anxiety, depression, fatigue, and physical activity after CA, and American Heart Association guidelines similarly recommend multimodal rehabilitation assessment and treatment for cognitive, physical, neurological, and cardiopulmonary impairments after CA.^9^, ^10^ These aspects of health have been explored after conventional CA.^9^ However, only a few small ECPR studies have assessed health-related quality of life, individual studies have reported cognitive function or symptoms of anxiety and depression, and none of these has assessed fatigue.^7^, ^11^, ^12^, ^13^, ^14^, ^15^

Functional outcomes quantify ability, but not how survivors value their lives. Life satisfaction captures this dimension – a global subjective evaluation that integrates physical, psychological and social dimensions into a single judgement.^16^ This is particularly relevant after ECPR, where circulation is often restored only after prolonged cardiopulmonary resuscitation, so that even a favourable functional outcome may not reflect meaningful recovery from the survivor's perspective.^17^, ^18^ To our knowledge, life satisfaction has not previously been assessed in ECPR survivors. Whether the ERC-recommended aspects of health relate to how these survivors evaluate their lives is unknown, and several of them are potentially modifiable targets for rehabilitation.

Therefore, the aim of this study was to evaluate life satisfaction and self-rated health in long-term ECPR survivors, and to assess their associations with functional outcome, cognitive function, anxiety, depression, fatigue and physical activity.

## Methods

### Study design and setting

This cross-sectional study included long-term survivors of ECPR at Sahlgrenska University Hospital, Gothenburg, Sweden. All patients received standard Advanced Cardiac Life Support (ACLS) and neuroprognostication according to ERC guidelines. The local ECPR programme and selection criteria have been published previously.^19^ The study was approved by the Regional Ethics Committee of Gothenburg and the Swedish Ethical Review Authority (Dnr 288-17 and 2020-02043) and was registered at ClinicalTrials.gov (NCT04198792). All participants provided written informed consent; for medical-record data on non-participants, informed consent was waived. The study is reported in accordance with the Strengthening the Reporting of Observational Studies in Epidemiology (STROBE) guidelines.^20^

### Participants

All ECPR survivors between January 2010 and October 2021, identified from the institutional ECPR database, were assessed for eligibility.^19^ Inclusion criteria were age ≥16 years and ≥1 year survival after ECPR. All eligible patients were invited by mail and telephone. Non-Swedish-speaking survivors received written information translated into their native language. All consenting survivors were included, and no sample size calculation was performed. Data on age at CA and mRS at 1 year for non-participants were obtained from medical records and have been published previously.^19^ The study was conducted between March 2022 and March 2023 at Sahlgrenska University Hospital or, for participants unable to attend the hospital, in their homes.

### Study outcomes

The primary outcomes were life satisfaction and self-rated health. Secondary outcomes were functional outcome, cognitive function, anxiety, depression, fatigue and physical activity level, the aspects of health recommended in the ERC guidelines for post-CA follow-up. In addition to their descriptive value, secondary outcomes were analysed for their associations with the primary outcomes.

### Outcome measures

#### Primary outcomes

Life satisfaction was assessed using a single question from the World Values Survey: “All things considered, how satisfied are you with your life as a whole these days?”, with responses ranging from 1 (completely dissatisfied) to 10 (completely satisfied).^21^, ^22^, ^23^

Self-rated health was assessed using the EuroQol 5-dimension 5-levels visual analogue scale (EQ-5D-5L VAS) from the EQ-5D-5L questionnaire, on which respondents rate their current overall health on a scale from 0 (worst imaginable health) to 100 (best imaginable health).^24^, ^25^, ^26^, ^27^

#### Secondary outcomes

Functional outcome was assessed using mRS, ranging from 0 (no symptoms) to 6 (death), where scores of 0–3 indicate no to moderate disability and define favourable outcome.^28^, ^29^

Cognitive function was screened using the Montreal Cognitive Assessment (MoCA), ranging from 0 to 30 points, with higher scores indicating better function. Scores <26 indicate a positive screen for cognitive impairment.^30^, ^31^

The Hospital Anxiety and Depression Scale (HADS) was used to screen for anxiety (HADS-A) and depression (HADS-D), each ranging from 0 to 21, with higher scores indicating more severe symptoms. Scores ≥8 indicate symptoms of mild and scores ≥11 symptoms of moderate anxiety or depression.^32^, ^33^, ^34^

Fatigue was assessed using the Mental Fatigue Scale (MFS), ranging from 0 to 42 points, with higher scores indicating greater fatigue.^35^ Scores ≥10.5 indicate at least mild mental fatigue.^36^, ^37^

Physical activity level was assessed with a question developed for the Targeted Temperature Management 2 (TTM2) trial and included in the ERC guidelines.^9^, ^38^ Participants reported the number of days in the preceding week with ≥30 min of moderate-intensity activity (e.g. brisk walking, cycling – sufficient to increase heart rate and breathing while still allowing conversation) and days with ≥20 min of vigorous-intensity activity. Participants were classified as adherent to European Society of Cardiology (ESC) and American Heart Association (AHA) cardiovascular prevention guidelines if they reported ≥150 min/week of moderate, ≥75 min/week of vigorous activity, or an equivalent combination.^39^, ^40^

### Additional outcome measures

Employment status before and after CA was assessed by self-report and categorised as employed full-time, part-time, disability pension/unemployed, or retired. Time from CA to return to work was recorded. Physical ability was further assessed using the 6-minute walk test (6-MWT), assessing functional exercise capacity and timed-stands test (TST), assessing lower limb functional strength.^41^, ^42^ Patients' own perceptions of mental recovery and dependency in daily activities were assessed using the questionnaire Two Simple Questions (TSQ).^43^, ^44^ In addition to the EQ-5D-5L VAS, the EQ-5D-5L index value was calculated using Swedish time trade-off (TTO) value sets, ranging from −0.31 (worst health status) to 1 (best possible health status).^45^, ^46^ Health-related quality of life was also assessed using the Short Form Health Survey (SF-36).^47^

### Demographic and clinical data

Demographic and clinical data, including living situation, marital status and educational level, were collected through structured interviews. History of neurological or psychiatric disease was obtained from interviews and medical records. Data regarding CA characteristics, hospital course, and prior assessments were obtained from the ECPR database.^19^ For participants whose native language was not Swedish, study assessments were conducted with assistance of professional translators.

### Statistical analysis

Continuous data are presented as median with interquartile range [IQR] and categorical data as *n* (%). Differences between participants and non-participants were assessed using Mann-Whitney *U* test.

Univariable linear regression was used to assess associations of baseline characteristics and secondary outcomes with the two primary outcomes (life satisfaction and EQ-5D-5L VAS). Two exploratory multivariable linear regression models were then fitted, one for each primary outcome. All secondary outcomes were entered simultaneously as continuous variables, and models were adjusted for sex, age at follow-up, and time from CA to follow-up, which were pre-specified as potential confounders. Normality of residuals was assessed, and the variance inflation factor (VIF) was used to assess collinearity. Two types of sensitivity analyses were performed to assess robustness. First, given the number of predictors relative to the sample size, reduced models were fitted including only variables significantly associated with the primary outcome in univariable analysis, with HADS-A and HADS-D combined into a single HADS-total score to reduce model complexity. Second, the main physical activity measure was replaced with the two alternative measures of physical function listed in the 2025 ERC guidelines – the EQ-5D-5L usual activities domain and the SF-36 physical function domain – to assess consistency. Differences in life satisfaction and EQ-5D-5L VAS across participants categorised by number of affected aspects of health (mRS, HADS-A, HADS-D, MoCA, and MFS beyond cut-off values) were assessed using the Kruskal-Wallis test. All tests were two-sided and *p* < 0.05 was considered statistically significant. Statistical analyses were performed using IBM SPSS Statistics (version 29; IBM Corp, Armonk, NY).

## Results

### Participants

Between 2010 and 2021, 131 patients were treated with ECPR at Sahlgrenska University Hospital. Of these, 34 were alive at study initiation, and 26 (76.5%) consented to participate (Fig. 1).

**Fig. 1:**
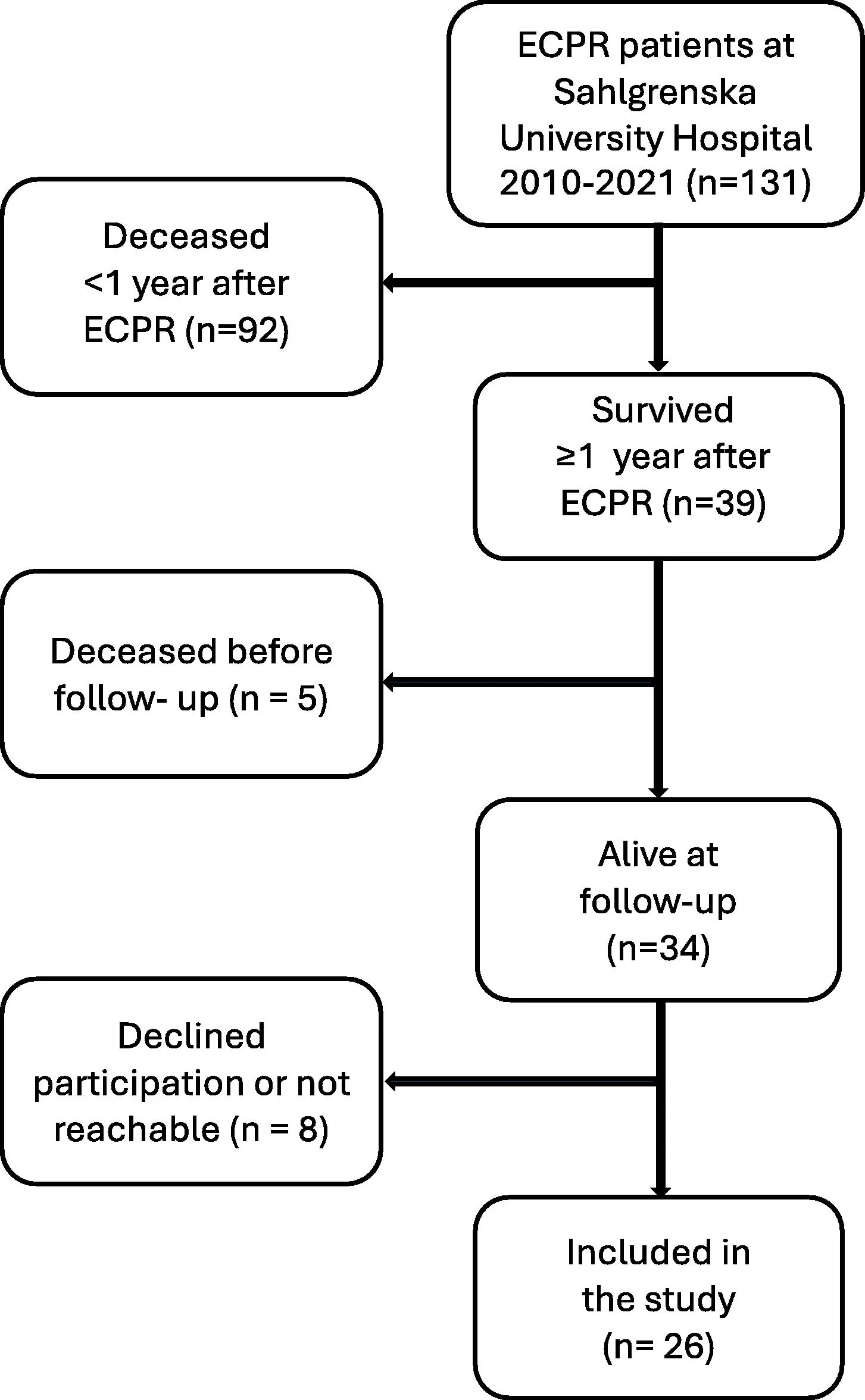
**Inclusion flowchart. Of 39 patients surviving ≥1 year after ECPR (extracorporeal cardiopulmonary resuscitation), 34 were alive at follow up and eligible for the study. Of those, 26 were included in the study**.

Six participants required professional interpreters for the assessment. There were no significant differences in age at ECPR or mRS at one year after ECPR between participants and eligible non-participants, nor between participants and the five patients who survived ≥1 year after ECPR but died before study initiation (Supplementary Table S1).

### Baseline characteristics

Participant characteristics are presented in Table 1. The median age at CA was 53.5 [43–57.5] years and 20 (76.9%) were male. Twelve participants (46.2%) had an out-of-hospital CA, and median CA duration was 40 [20–60] min. Ischaemic aetiology was the most common cause of CA (69.2%). Median intensive care unit stay was 19.5 [9–30] days and median total hospital stay was 52.5 [34.5–71] days. Median ventilator time was 13 [5.8–23.3] days and 14 (53.8%) had renal replacement therapy during intensive care unit stay.

**Table 1:** Baseline, cardiac arrest, and in-hospital characteristics.

**Pre-arrest characteristics**	**Overall cohort (*n* = 26)**
Age at CA, years	53.5 [43–57.5]
Sex, male	20 (76.9)
Hypertension	5 (19.2)
Diabetes mellitus	3 (11.5)
Stroke	2 (7.7)
History of psychiatric disorder^a^	8 (30.8)
Employed at CA	22 (84.6)
**Educational level**	
– No education	2 (7.7)
– Primary and lower secondary education	1 (3.8)
– Upper secondary education	11 (42.3)
– Post secondary education <3 years	2 (7.7)
– Post secondary education ≥3 years	10 (38.5)
Swedish as native language	19 (73.1)
**Cardiac arrest characteristics**	
OHCA	12 (46.2)
Initial shockable rhythm	12 (46.2)
Total cardiac arrest time, minutes^b^	40 [20–60]
**Aetiology**	
– Ischaemic	18 (69.2)
– Cardiomyopathy	4 (15.4)
– Myocarditis	1 (3.8)
– Other (hypothermia, arrhythmia)	3 (11.5)
Accidental hypothermia	2 (7.7)
Coronary angiogram	17 (65.4)
Percutaneous coronary intervention	15 (57.7)
rSO_2_ (INVOS) (*n* = 10)	26 [20–35]
Pre-ECPR score (*n* = 24)	0.49 [0.3–0.7]
**In-hospital characteristics**	
Days on ECMO	2 [2–6]
Days on ventilator	13 [5.8–23.3]
Length of ICU stay, days	19.5 [9–30]
Length of hospital stay, days	52.5 [34.5–71]
In-hospital rehabilitation	10 (38.5)
Renal replacement therapy	14 (53.8)
Delirium^c^	14 (53.8)
Neurological insult during ICU^d^	11 (42.3)
Heart transplantation	7 (26.9)
**Ventricular assist device**	
– Excor	3 (11.5)
– HeartMate	1 (3.8)
Cardiac rehabilitation	16 (61.5)
Neurorehabilitation	12 (46.2)

Continuous data are presented as median [interquartile range] and categorical data as number (%).

*Abbreviations:* CA, cardiac arrest; CT, computed tomography; ECMO, extracorporeal membrane oxygenation; ECPR, extracorporeal cardiopulmonary resuscitation; ICU, intensive care unit; OHCA, out-of-hospital cardiac arrest; rSO_2_, regional cerebral oxygen saturation.

a History of psychiatric disorder includes any type of depression, anxiety, exhaustion disorder, post-traumatic stress disorder or other psychiatric disorder mentioned in the medical record.

b Total cardiac arrest time is cumulative time from cardiac arrest to ECMO initiation, with time for intermittent ROSC subtracted.

c Delirium was defined by either a registered diagnosis or described in text in the medical record during ICU stay.

d Neurological insult was defined as seizures, haemorrhages or ischaemic lesion described on CT scan or neurological deficits described in text in the medical record during ICU stay.

### Primary outcomes

At a median follow-up of 5 [2–7] years after ECPR, the median life satisfaction score was 8 [6–9] out of 10. Self-rated health (EQ-5D-5L VAS) was 81 out of 100 [70–90] (Table 2).

**Table 2:** Long-term follow-up outcomes.

**Outcome**	**Overall cohort (*n* = 26)**
Follow-up time, years	5 [2–7]
**Primary outcomes**	
Life satisfaction at follow-up	8 [6–9]
EQ-5D-5L VAS	81 [70–90]
**Secondary outcomes**	
mRS	1.5 [0–2]
– Favourable outcome (mRS 0–3)	25 (96)
MoCA (*n* = 25)	25 [23–27]
– Positive cognitive screen (MoCA < 26)	14 (56)
HADS anxiety	5 [1–10]
– Symptoms of at least mild anxiety (HADS-A ≥ 8)	11 (42.3)
– Symptoms of at least moderate anxiety (HADS-A ≥ 11)	4 (15.4)
HADS depression	2 [1–4]
– Symptoms of at least mild depression (HADS-D ≥ 8)	4 (15.4)
– Symptoms of at least moderate depression (HADS-D ≥ 11)	0 (0)
MFS	10.3 [3.5–16.5]
– Fatigue (MFS ≥ 10.5)	13 (50)
Moderate physical activity^a^ days/week	7 [4–7]
Vigorous physical activity^b^ days/week	2 [0–3]
Did not meet physical activity recommendation^c^	5 (19.2)

**Additional outcomes**	
Employed before cardiac arrest (*n* = 22)	
– Return to full time	14 (63.6)
– Return to part time	2 (9.0)
– Disability pension/unemployed	5 (22.7)
– Retired	1 (4.5)
Time to return to work, months	8.5 [6–16.5]
Two simple questions – help needed	3 (11.5)
– If help is needed, is this new after CA	3 (11.5)
Two simple questions – mentally recovered	9 (34.6)
6-min walk test, m (*n* = 20)	535 [465–573]
Timed-stands test, s (*n* = 24)	17.8 [14.4–23.0]
EQ-5D-5L TTO	0.89 [0.83–0.94]
SF-36 Physical function (*n* = 25)	90 [80–95]
SF-36 Role physical (*n* = 24)	100 [25–100]
SF-36 Bodily pain (*n* = 25)	100 [78.5–100]
SF-36 General health (*n* = 25)	65 [45–77.5]
SF-36 Vitality (*n* = 25)	80 [52.5–85]
SF-36 Social function (*n* = 25)	87 [75–100]
SF-36 Role emotional (*n* = 24)	100 [41.3–100]
SF-36 Mental health (*n* = 25)	84 [58–94]

Continuous data presented as median [IQR] and categorical as number (%). The SF-36 domains are presented as a spider plot of median scores in Supplements Fig. S1.

*Abbreviations:* CA, cardiac arrest; EQ-5D-5L, EuroQol 5-dimension 5-levels; HADS, Hospital Anxiety and Depression Scale; MFS, Mental Fatigue Scale; MoCA, Montreal Cognitive Assessment; mRS, modified Rankin Scale; SF-36, Short Form Health Survey; TTO, time trade-off; VAS, visual analogue scale.

a Days per week with ≥30 min of moderate-intensity physical activity.

b Days per week with ≥20 min of vigorous physical activity.

c Defined as <150 min/week of moderate-intensity activity, <75 min/week of vigorous-intensity activity, or no equivalent combination.

### Secondary outcomes

Functional outcome was favourable (mRS 0–3) in 25 participants (96%). A positive screen for cognitive impairment (MoCA < 26) was present in 14 participants (56%). Symptoms of anxiety (HADS-A ≥ 8) were present in 11 (42%), and symptoms of depression (HADS-D ≥ 8) in four (15%). Mental fatigue (MFS ≥ 10.5) was present in 13 (50%). Participants reported a median of 7 [4–7] days with ≥30 min of moderate physical activity and 2 [0–3] days of vigorous physical activity in the preceding week. Five participants (19%) did not meet the ESC/AHA recommendation of physical activity.

### Additional outcomes

All participants but one were living at home at follow-up. Among the 22 participants employed before CA, 16 (73%) had returned to work at a median of 8.5 [6–16.5] months.

### Associations with life satisfaction and self-rated health

In univariable analyses, life satisfaction was positively associated with moderate physical activity and negatively associated with mRS, HADS-A and HADS-D (Fig. 2, Table 3). EQ-5D-5L VAS was positively associated with physical activity and negatively with mRS.

**Fig. 2:**
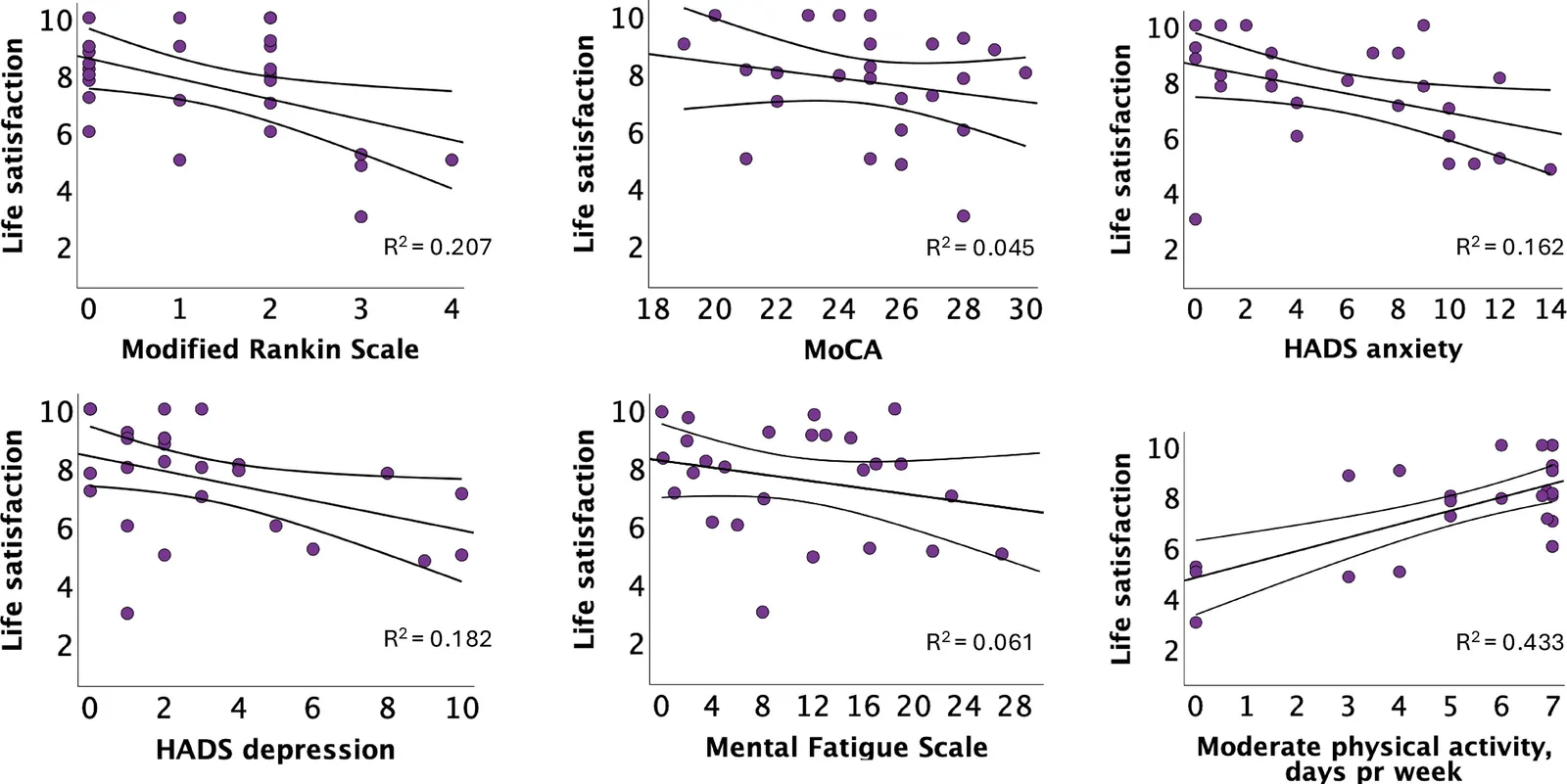
**Univariable associations between life satisfaction and functional outcome (mRS), cognitive screening score (MoCA), symptoms of anxiety (HADS-A) and depression (HADS-D), mental fatigue (MFS) and moderate physical activity (days/week). Lines represent linear regression estimates with 95% confidence intervals. Regression estimates with corresponding statistics are reported in**Table 3. *Abbreviations:* HADS, Hospital Anxiety and Depression Scale; MFS, Mental Fatigue Scale; MoCA, Montreal Cognitive Assessment; mRS, modified Rankin Scale.

**Table 3:** Associations of secondary outcomes with life satisfaction and self-rated health.

**Variable**	**Life satisfaction**				**EQ-5D-5L VAS**			
	**Univariable**		**Multivariable**		**Univariable**		**Multivariable**	
	***B* (95% CI)**	***p***	***B* (95% CI)**	***p***	***B* (95% CI)**	***p***	***B* (95% CI)**	***p***
mRS	−0.70 (−1.29, −0.11)	0.022	−0.29 (−1.09, 0.51)	0.459	−9.22 (−15.76, −2.67)	0.008	−3.66 (−13.80, 6.48)	0.455
MoCA	−0.13 (−0.39, 0.13)	0.313	−0.11 (−0.33, 0.11)	0.318	−0.61 (−3.51, 2.30)	0.670	0.25 (−2.54, 3.04)	0.850
HADS anxiety	−0.17 (−0.33, −0.01)	0.038	−0.05 (−0.28, 0.18)	0.641	−1.29 (−3.25, 0.66)	0.185	0.27 (−2.62, 3.15)	0.846
HADS depression	−0.25 (−0.48, −0.01)	0.040	−0.09 (−0.36, 0.19)	0.522	−1.61 (−4.50, 1.29)	0.263	0.48 (−3.03, 3.99)	0.775
MFS	−0.06 (−0.16, 0.04)	0.214	0.06 (−0.09, 0.20)	0.431	−0.90 (−2.01, 0.22)	0.109	−0.09 (−1.97, 1.78)	0.918
Moderate physical activity days/week	0.52 (0.27, 0.77)	<0.001	0.36 (0.06, 0.67)	0.023	6.04 (3.14, 8.93)	<0.001	4.60 (0.71, 8.50)	0.024

Multivariable analyses are adjusted for age, sex and time between cardiac arrest and follow-up. One missing MoCA value was imputed for the multivariable analyses using the cohort median.

*Abbreviations:* EQ-5D-5L, EuroQol 5-dimension 5-levels; HADS, Hospital Anxiety and Depression Scale; MFS, Mental Fatigue Scale; MoCA, Montreal Cognitive Assessment; mRS, modified Rankin Scale; VAS, visual analogue scale.

In exploratory multivariable analyses, moderate physical activity was the only variable associated with life satisfaction (*B* 0.36 per day/week; 95% confidence interval (CI) 0.06–0.67; *p* = 0.023) and EQ-5D-5L VAS (*B* 4.60; 95% CI 0.71–8.50; *p* = 0.024) (Table 3). Model diagnostics showed normally distributed residuals and no multicollinearity (VIF < 4).

Two types of sensitivity analyses were performed to assess robustness of the main exploratory finding, with the results largely consistent across model specifications and related physical activity measures. First, reduced models including only variables significantly associated with the primary outcomes in univariable analyses were fitted. In these models, moderate physical activity was the only predictor that remained associated with both life satisfaction (*B* 0.45; 95% CI 0.17–0.74; *p* = 0.003) and EQ-5D-5L VAS (*B* 4.94; 95% CI 1.62–8.26; *p* = 0.005) (Supplementary Table S2). Second, to assess consistency across related measures, the main physical activity measure was replaced with the two alternative measures listed in the ERC guidelines. SF-36 physical function domain was associated with both primary outcomes, whereas the EQ-5D-5L usual activities domain was associated with life satisfaction but not significantly with EQ-5D-5L VAS (*B* −11.75; 95% CI −23.76–0.26; *p* = 0.055) (Supplementary Tables S3 and S4). Exploratory univariable associations between the participant characteristics and the primary outcomes are shown in Supplementary Tables S5 and S6 for descriptive purposes.

There was no significant difference in life satisfaction (*H*(4) = 3.85, *p* = 0.427) or EQ-5D-5L VAS (*H*(4) = 4.63, *p* = 0.328) across the number of affected aspects of health (functional outcome, cognition, anxiety, depression, and fatigue) (Fig. 3).

**Fig. 3:**
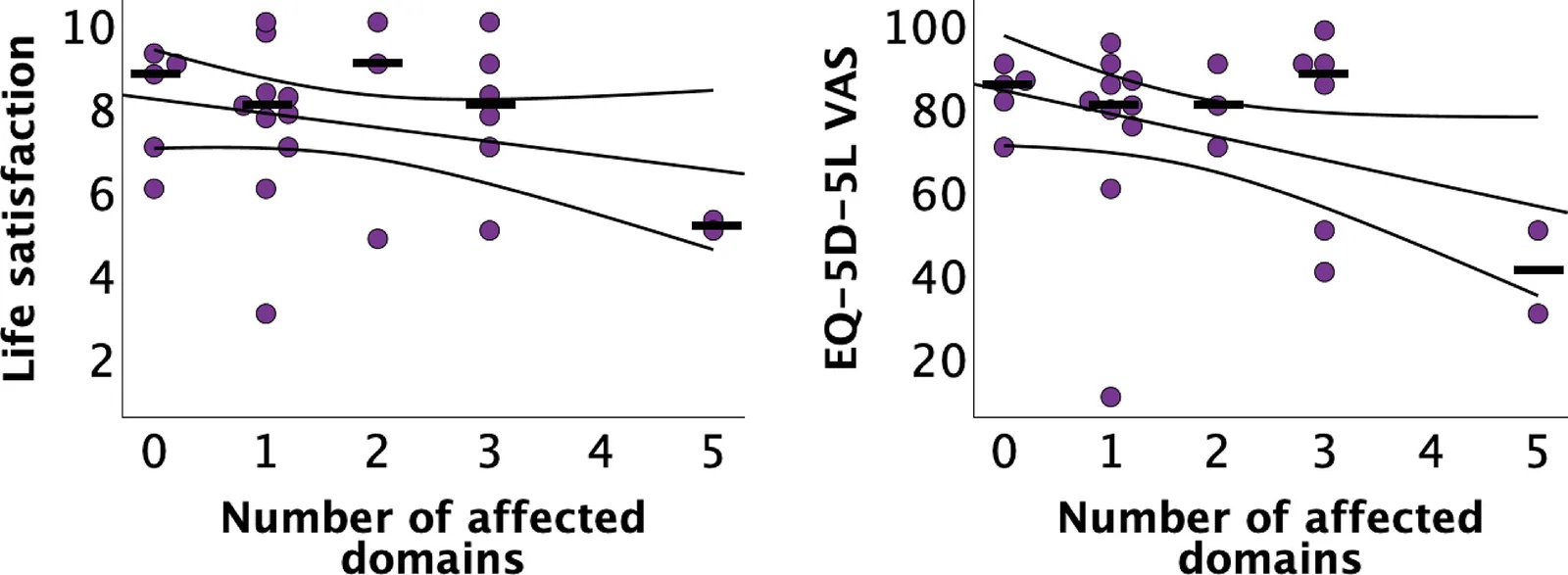
**Number of affected domains in relation to (A) life satisfaction and (B) self-rated health (EQ-5D-5L VAS). A domain was counted as affected if the score was beyond its cut-off: functional outcome (mRS > 3), cognition (MoCA < 26), anxiety (HADS-A ≥ 8), depression (HADS-D ≥ 8) and fatigue (MFS ≥ 10.5). Median values are shown for each group. Differences across the number of affected domains were tested with the Kruskal-Wallis test, and no significant difference in life satisfaction or self-rated health was found**. *Abbreviations:* EQ-5D-5L, EuroQol 5-dimension 5-level; HADS, Hospital Anxiety and Depression Scale; MFS, Mental Fatigue Scale; MoCA, Montreal Cognitive Assessment; mRS, modified Rankin Scale.

## Discussion

At a median of 5 years after ECPR, survivors reported life satisfaction and self-rated health similar to population reference values, despite frequent positive screens for cognitive impairment, symptoms of anxiety, and fatigue.^21^, ^27^ In exploratory multivariable analyses, moderate physical activity was the only variable associated with the outcomes.

After ECPR, where prolonged CA and extracorporeal membrane oxygenation (ECMO) treatment may leave survivors with physical, cognitive or emotional sequelae, life satisfaction might be expected to be lower than in the general population. Yet, in our cohort, despite a median CA duration of 40 min and hospital stays exceeding 50 days, life satisfaction was high, median of 8 out of 10. This is descriptively similar to that of the general Swedish population in the 2011 World Values Survey, where the same single-item life satisfaction question yielded a mean score of 7.55.^21^ It is also consistent with the TTM2 trial where fit and prefrail survivors reported median life satisfaction of 7–8 on the same question, at 6 and 24 months after CA.^23^ Similarly, Wachelder et al. found no difference in LiSat-9 scores between CA survivors and patients with myocardial infarction without CA.^48^ Although median life satisfaction was high, these findings should not be interpreted as uniformly good subjective outcome. One participant reported a life satisfaction score of 3 and four reported scores of 5, indicating that a minority of survivors experienced substantially lower life satisfaction.

Median self-rated health (EQ-5D-5L VAS) was 81, similar to Swedish population norms of 80.^27^ Prior ECPR studies report EQ-5D-5L VAS of 52.5 in 33 German patients,^15^ 70 in 15 Danish patients,^7^ and 80 in 24 patients from Australia and New Zealand.^14^ The reasons for these differences are unclear; small samples and differing case mix may contribute.

Positive screens for cognitive impairment, anxiety, depression and fatigue were frequent but need to be read against population norms. A positive MoCA screen in 56% is similar to population-based data, in which half or more of adults in this age group score below the cut-off.^49^, ^50^ In ECPR, Rasalingam Mørk et al. reported similar findings to ours with a mean MoCA of 26.4.^13^ Symptoms of anxiety were present in 42%, exceeding both population norms (21%) and conventional CA frequencies (22–28%).^51^, ^52^ Symptoms of depression were present in 15%, similar to general population and conventional CA frequencies.^52^, ^53^ In ECPR, Gregers et al. reported a similar pattern using the same instrument, with anxiety in 40% and depression in 7% in a cohort of 15 survivors.^7^ Fatigue was present in 50%, markedly exceeding healthy controls.^36^, ^54^ Fatigue is common also in conventional CA survivors, though comparisons are limited by different instruments.^55^, ^56^ To our knowledge, no prior study has assessed fatigue in ECPR survivors. Thus, while cognition and depression appear similar to the general population, anxiety and fatigue were more prevalent among ECPR survivors.

The number of positive screens showed no clear relationship with life satisfaction or self-rated health (Fig. 3). Higher mRS, anxiety and depression scores were associated with lower life satisfaction in univariable analyses, but these associations were attenuated and imprecise after adjustment. Thus, although mRS is the standard measure of favourable outcome after CA, our findings suggest that functional status alone does not fully capture life satisfaction after ECPR. It captures functional ability, but not how survivors evaluate their lives.

Several factors may explain why scores beyond established cut-offs did not translate into lower subjective well-being. For ECPR survivors, simply being alive may redefine what matters – a response shift in which internal standards and values are recalibrated following life-threatening illness.^57^ Qualitative studies have described CA survivors as continuing life in a more conscious and meaningful way, and this sense of gratitude for a life that was nearly lost may overshadow residual difficulties.^58^ Survivors may develop coping strategies, adapting routines to accommodate limitations, thereby maintaining a high quality of everyday life.

By contrast, moderate physical activity was associated with both life satisfaction and self-rated health in multivariable analysis. This association was consistent in sensitivity analyses using the EQ-5D-5L usual activities and SF-36 physical function domains.^9^ The association is biologically plausible, and physical activity may also enable social participation, which could in itself contribute to well-being.^59^, ^60^ However, whether the lower life satisfaction and self-rated health seen in less active survivors reflects their reduced activity, or whether reduced activity reflects their lower well-being – or both – cannot be determined from our cross-sectional data. Evidence from the general population suggests a bidirectional association.^61^

Regardless of direction, physical activity is a relevant component of post-ECPR follow-up assessment, and it is potentially modifiable. Whether promoting it enhances well-being after ECPR remains to be tested, but exercise interventions have been associated with improvements in health-related quality of life in stroke survivors.^62^ Beyond well-being, physical activity is also central to reduce the risk of recurrent cardiovascular events and all-cause mortality.^39^, ^40^ In our cohort 19% did not reach this recommendation.

Return to work reflects complex functional recovery and societal reintegration. Among previously employed participants, 73% had returned to work after a median of 8.5 months. The proportion is within the 51–79% reported after ECPR.^13^, ^14^, ^63^ One ECPR study reported a median time to return of 6.5 months.^63^ A Danish nationwide cohort after conventional CA found that 77% had returned after a median of 4 months (interquartile range 1–19).^64^

Long-term outcome data after ECPR remain sparse, and this study contributes to a limited evidence base. Strengths include a long follow-up period (median five years), comprehensive assessment of the aspects of health recommended by the ERC guidelines using, a high participation rate (76.5%), and the inclusion of life satisfaction as a primary outcome. Limitations include the single-centre design and sample size, which limit generalisability, and the cross-sectional assessment, which precludes causal inference. The findings describe long-term survivors and do not apply to ECPR patients overall. Within this group, participants did not differ significantly in age or mRS from non-participants or from survivors who died before inclusion, although these comparisons involve small numbers. The number of predictors relative to the sample size is an important limitation of the exploratory multivariable analyses, which makes the estimates unstable and limits confidence in which variables remain statistically significant. The predictors nevertheless comprised all ERC-recommended follow-up aspects of health rather than a data-derived selection, and the association between moderate physical activity and both primary outcomes was consistent in direction across the full model, reduced models and the two physical function measures listed in the ERC guidelines.^9^ The models are presented as hypothesis-generating and require confirmation in larger cohorts. Physical activity was self-reported, but the finding was consistent across the three measures of physical activity and function. Population comparisons were descriptive and were not matched or adjusted for sex, socioeconomic status, comorbidity, or survival selection.

## Conclusion

At long-term follow-up after ECPR, survivors reported life satisfaction and self-rated health similar to population reference values, while anxiety and fatigue were more frequent. In exploratory multivariable analyses, moderate physical activity was associated with both life satisfaction and self-rated health, whereas the association with functional outcome (mRS) was attenuated and imprecise after adjustment. These findings support the assessment of life satisfaction and physical activity in post-ECPR follow-up. Because the design was cross-sectional, the direction of this association cannot be determined, and whether physical activity interventions can improve well-being after ECPR remains to be tested.

## Declaration of generative AI and AI-assisted technologies in the manuscript preparation process

During the preparation of this work the authors used Claude and ChatGPT in order to improve language. After using this tool the authors reviewed and edited the content as needed and take full responsibility for the content of the published article.

## CRediT authorship contribution statement

**Anna Henningsson:** Writing – original draft, Visualization, Methodology, Investigation, Formal analysis, Data curation. **Pia Watson:** Investigation, Data curation, Conceptualization. **Magnus Segerström:** Writing – review & editing, Conceptualization. **Petra Redfors:** Writing – review & editing, Methodology, Conceptualization. **Bengt Redfors:** Writing – review & editing, Visualization, Supervision, Methodology, Investigation, Funding acquisition, Formal analysis, Data curation, Conceptualization.

## Funding

This work was supported by the Swedish Heart-Lung Foundation [grant number 20200197], the Swedish Society of Medicine [grant number SLS-935423], the Gothenburg Medical Society [grant number GLS-1021474] and the Swedish state under the agreement between the Swedish Government and the country councils, the ALF agreement (ALFGBG-1006815). The funders had no role in study design, data collection, analysis or interpretation, writing of the manuscript, or the decision to submit the article for publication.

## Declaration of competing interest

The authors declare the following financial interests/personal relationships which may be considered as potential competing interests: Bengt Redfors reports financial support was provided by Swedish Society of Medicine. Bengt Redfors reports financial support was provided by The Swedish Heart Lung Foundation. Anna Henningsson reports financial support was provided by Gothenburg medical society. Bengt Redfors reports financial support was provided by Swedish state under the agreement between the Swedish Government and the country councils, the ALF agreement. If there are other authors, they declare that they have no known competing financial interests or personal relationships that could have appeared to influence the work reported in this paper.

## Appendix A Supplementary material

Supplementary Data 1 The supplementary material includes data on age and Modified Rankin Scale (mRS) for non-participants, sensitivity analysis with SF-36 physical function and EQ-5D-5L usual activity instead of days pr week with moderate physical activity, exploratory univariable analysis of participant characteristics and follow-up data, and spider plot of median SF-36 scores.
