# Supplementary material for: Life satisfaction and self-rated health in long-term survivors after extracorporeal cardiopulmonary resuscitation (ECPR): associations with functional outcome, cognition, anxiety, depression, fatigue, and physical activity
Source: Resusc Plus. 2026 Oct 7;32:101521. doi: 10.1016/j.resplu.2026.101521 (PMC13717885; doi:10.1016/j.resplu.2026.101521)
Supplement: Supplementary file 1 [file mmc1.docx]

**Supplementary document**

|  | Participants (n=26) | Non-participating (n=8) | P | Deceased before  follow up (n=5) | P |
| --- | --- | --- | --- | --- | --- |
| Age at ECPR | 53.5 [44, 57] | 41 [33, 60] | 0.307 | 39 [27, 39] | 0.115 |
| mRS ≥1 year after ECPR | 1.5 [0, 2] | 0 [0, 2] | 0.368 | 2 [2, 3] | 0.115 |

**Table S1.** Age at ECPR and mRS ≥1 year after ECPR.

Differences between participants and non-participants were evaluated using the Mann-Whitney U-test. Median values and [IQR] are presented.

Abbreviations: ECPR, extracorporeal cardiopulmonary resuscitation; mRS, Modified Rankin Scale.

**Table S2.** Sensitivity analysis with univariable statistically significant parameters for primary outcomes.

| **Variable** | **Life Satisfaction** | | | | **EQ-5D-5L VAS** | | | |
| --- | --- | --- | --- | --- | --- | --- | --- | --- |
|  | **Univariable** | | **Multivariable** | | **Univariable** | | **Multivariable** | |
|  | **B (95% CI)** | **p** | **B (95% CI)** | **p** | **B (95% CI)** | **p** | **B (95% CI)** | **p** |
| mRS | –0.70  (–1.29, –0.11) | 0.022 | 0.03  (–0.62, 0.68) | 0.927 | –9.22  (–15.76, –2.67) | 0.008 | –4.24  (–10.80, 2.31) | 0.193 |
| HADS  total | –0.13  (–0.23, –0.02) | 0.018 | –0.09  (–0.19, 0.02) | 0.103 |  |  |  |  |
| Days with >30 min physical activity/ week | 0.52  (0.27, 0.77) | <0.001 | 0.45  (0.17, 0.74) | 0.003 | 6.04  (3.14, 8.93) | <0.001 | 4.94  (1.62, 8.26) | 0.005 |

To reduce the number of variables, the HADS depression and anxiety subscales was combined into one single variable (HADS total). This combined score represents a measure of general psychological distress.

Abbreviations: EQ-5D-5L, EuroQol 5-dimension 5-levels; HADS Hospital Anxiety and Depression Scale; mRS, modified Rankin Scale; VAS, visual analog scale.

**Table S3.** Sensitivity analysis including parameter “SF-36 physical function”, instead of parameter “moderate physical activity days/week.

| **Variable** | **Life Satisfaction** | | | | **EQ-5D-5L VAS** | | | |
| --- | --- | --- | --- | --- | --- | --- | --- | --- |
|  | **Univariable** | | **Multivariable** | | **Univariable** | | **Multivariable** | |
|  | **B (95% CI)** | **p** | **B (95% CI)** | **p** | **B (95% CI)** | **p** | **B (95% CI)** | **p** |
| mRS | –0.70  (–1.29, –0.11) | 0.022 | –0.14  (–0.79, 0.51) | 0.652 | –9.22  (–15.76, –2.67) | 0.008 | –1.76  (–10.49, 6.98) | 0.674 |
| MoCA | –0.13  (–0.39, 0.13) | 0.313 | –0.18  (–0.35, –0.01) | 0.044 | –0.61  (–3.51, 2.30) | 0.684 | –0.66  (–2.99, 1.66) | 0.552 |
| HADS Anxiety | –0.17  (–0.33, –0.01) | 0.038 | –0.09  (–0.28, 0.09) | 0.307 | –1.29  (–3.25, 0.66) | 0.185 | –0.28  (–2.75, 2.19) | 0.814 |
| HADS Depression | –0.248  (–0.48, –0.012) | 0.040 | –0.10  (–0.32, 0.12) | 0.333 | –1.61  (–4.50, 1.29) | 0.263 | 0.27  (–2.68, 3.23) | 0.846 |
| MFS | –0.06  (–0.16, 0.04) | 0.214 | 0.11  (–0.01, 0.24) | 0.067 | –0.90  (–2.01, 0.22) | 0.109 | 0.64  (–0.99, 2.26) | 0.417 |
| SF–36 physical function | 0.05  (0.03, 0.07) | <0.001 | 0.05  (0.02, 0.07) | <0.001 | 0.59  (0.40, 0.79) | <0.001 | 0.61  (0.28, 0.95) | 0.001 |

Multivariable analyses are adjusted for age, sex and time between cardiac arrest and follow–up. One missing MoCA value was imputed for the multivariable analyses using the cohort median.

Abbreviations: SF-36, Short-Form Health Survey; HADS, Hospital Anxiety and Depression Scale; MFS, Mental Fatigue Scale; MoCA, The Montreal Cognitive Assessment; mRS, modified Rankin Scale; VAS, visual analog scale.

**Table S4.** Sensitivity analysis including parameter “EQ-5D-5L, usual activity “instead of parameter “moderate physical activity days/week”.

| **Variable** | **Life Satisfaction** | | | | **EQ-5D-5L VAS** | | | |
| --- | --- | --- | --- | --- | --- | --- | --- | --- |
|  | **Univariable** | | **Multivariable** | | **Univariable** | | **Multivariable** | |
|  | **B (95% CI)** | **p** | **B (95% CI)** | **p** | **B (95% CI)** | **p** | **B (95% CI)** | **p** |
| mRS | –0.70  (–1.29, –0.11) | 0.022 | 0.24  (–0.69, 1.17) | 0.594 | –9.22  (–15.76, –2.67) | 0.008 | 0.31  (–12.82, 13.44) | 0.961 |
| MoCA | –0.13  (–0.39, 0.13) | 0.313 | –0.04  (–0.26, 0.18) | 0.709 | –0.61  (–3.51, 2.30) | 0.684 | 0.78  (–2.30, 3.86) | 0.599 |
| HADS Anxiety | –0.17  (–0.33, –0.01) | 0.038 | –0.10  (–0.31, 0.12) | 0.357 | –1.29  (–3.25, 0.66) | 0.185 | –0.14  (–3.21, 2.93) | 0.922 |
| HADS Depression | –0.25  (–0.48, –0.012) | 0.040 | –0.16  (–0.42, 0.10) | 0.207 | –1.61  (–4.50, 1.29) | 0.263 | –0.33  (–4.04, 3.38) | 0.851 |
| MFS | –0.06  (–0.16, 0.04) | 0.214 | 0.09  (–0.05, 0.23) | 0.177 | –0.90  (–2.01, 0.22) | 0.109 | 0.29  (–1.69, 2.27) | 0.759 |
| EQ-5D-5L –  usual activity | –0.93  (–1.53, –0.33) | 0.004 | –1.21  (–2.06, –0.36) | 0.008 | –11.94  (–18.46, –5.42) | <0.001 | –11.75  (–23.76, 0.26) | 0.055 |

Multivariable analyses are adjusted for age, sex and time between cardiac arrest and follow–up. One missing MoCA value was imputed for the multivariable analyses using the cohort median.

Abbreviations: EQ-5D-5L, EuroQol 5-dimension 5-levels; HADS, Hospital Anxiety and Depression Scale; MFS, Mental Fatigue Scale; MoCA, The Montreal Cognitive Assessment; mRS, modified Rankin Scale; VAS, visual analog scale.

**Table S5.** Exploratory univariable analysis of participant characteristics and follow-up data in relation to life satisfaction, presented for descriptive purposes.

| **Life satisfaction** | | |
| --- | --- | --- |
| **Variable** | **B (95% CI)** | **p** |
| **Pre-arrest characteristics** |  |  |
| Age at CA, years | 0.030 (–0.032, 0.092) | 0.331 |
| Sex, male | 2.267 (0.690, 3.843) | 0.007 |
| Swedish as native language | 0.008 (–1.743, 1.758) | 0.993 |
| Hypertension | –0.962 (–2.890, 0.966) | 0.313 |
| Diabetes mellitus | –0.652 (–3.067, 1.763) | 0.582 |
| History of psychiatric disorder | –1.556 (–3.105, –0.006) | 0.049 |
| Education level |  | 0.501 |
| Employed at CA | –0.546 (–1.124, 0.033) | 0.063 |
| **Cardiac arrest characteristics** |  |  |
| OHCA | 0.452 (–1.094, 1.998) | 0.552 |
| Bystander CPR | 0.967 (–0.831, 2.764) | 0.278 |
| Shockable rhythms | –0.184 (–0.818, 0.450) | 0.555 |
| CA time, minutes | –0.016 (–0.037, 0.004) | 0.115 |
| Aetiology |  | 0.029 |
| Coronary angiogram | 0.882 (–0.707, 2.472) | 0.263 |
| Percutaneous coronary intervention | 0.370 (–1.194, 1.934) | 0.630 |
| rSO2 INVOS™ | –0.093 (–0.223, 0.036) | 0.135 |
| PreECPR score | 0.403 (–3.525, 4.332) | 0.833 |
| **In-hospital characteristics** |  |  |
| Days on ECMO | 0.019 (–0.090, 0.127) | 0.727 |
| Days on ventilator | –0.002 (–0.050, 0.047) | 0.949 |
| Length of ICU stay, days | –0.003 (–0.047, 0.042) | 0.906 |
| Length of hospital stay, days | –0.008 (–0.031, 0.015) | 0.467 |
| In hospital rehabilitation | 0.200 (–1.394, 1.794) | 0.798 |
| Renal replacement therapy | –0.940 (–2.447, 0.566) | 0.210 |
| ICU delirium | 0.143 (–1.414, 1.699) | 0.851 |
| Neurological insult during ICU | –0.370 (–1.934, 1.194) | 0.630 |
| Heart transplantation | 0.970 (–0.733, 2.672) | 0.251 |
| Accidental hypothermia | –1.708 (–4.532, 1.116) | 0.224 |
| LVAD | 0.716 (–0.432, 1.865) | 0.210 |
| Cardiac rehabilitation | 0.775 (–0.788, 2.338) | 0.316 |
| Neurorehabilitation | 0.167 (–1.390, 1.723) | 0.827 |
| **Physical characteristics** |  |  |
| Follow up time, years | 0.017 (–0.202, 0.236) | 0.874 |
| Age at follow up | 0.036 (–0.030, 0.101) | 0.270 |
| Living at home | –1.340 (–3.279, 0.599) | 0.167 |
| Marital status | 0.103 (–1.468, 1.674) | 0.893 |
| Employed at follow up | –0.335 (–0.705, 0.034) | 0.073 |
| Return to work | –0.289 (–1.545, 0.967) | 0.639 |
| Moderate physical activity days/week | 0.520 (0.267, 0.773) | <0.001 |
| Vigorous physical activity days/week | 0.371 (0.025, 0.717) | 0.036 |
| 6 minute walk test, meters | 0.004 (–0.003, 0.010) | 0.279 |
| 5x Sit to stand test, seconds | –0.085 (–0.158, –0.012) | 0.024 |
| Two Simple Questions – help needed | –2.913 (–5.011, –0.815) | 0.009 |
| Two Simple Questions – mentally recovered | 0.647 (–0.962, 2.257) | 0.415 |

No adjustment for multiple comparisons was made.

Abbreviations: CA, cardiac arrest; CI, confidence interval; CPR, cardiopulmonary resuscitation; ECMO, extracorporeal membrane oxygenation; ECPR, extracorporeal cardiopulmonary resuscitation; EQ-5D-5L, EuroQol 5-dimension 5-level; ICU, intensive care unit; LVAD, left ventricular assist device; OHCA, out-of-hospital cardiac arrest; rSO2, regional cerebral oxygen saturation; VAS, visual analogue scale.

**Table S6.** Exploratory univariable analysis of participant characteristics and follow-up data in relation to EQ-5D-5L VAS, presented for descriptive purposes.

| **EQ5D5L VAS** | | |
| --- | --- | --- |
| **Variable** | **B (95% CI)** | **p** |
| **Pre-arrest characteristics** |  |  |
| Age at CA, years | –0.165 (–0.894, 0.565) | 0.646 |
| Sex, male | 22.633 (3.684, 41.583) | 0.021 |
| Swedish as native language | 3.233 (–16.871, 23.337) | 0.743 |
| Hypertension | –18.667 (–39.937, 2.604) | 0.083 |
| Diabetes mellitus | –15.913 (–43.073, 11.247) | 0.238 |
| History of psychiatric disorder | –5.889 (–25.094, 13.316) | 0.533 |
| Educational level |  | 0.101 |
| Employed at CA | –4.253 (–11.190, 2.684) | 0.218 |
| **Cardiac arrest characteristics** |  |  |
| OHCA | 1.071 (–16.851, 18.994) | 0.903 |
| Bystander CPR | 3.133 (–18.039, 24.305) | 0.763 |
| Shockable rythm | –4.318 (–11.438, 2.802) | 0.223 |
| CA time, minutes | –0.161 (–0.404, 0.082) | 0.184 |
| Aetiology |  | 0.126 |
| Coronary angiogram | 3.176 (–15.563, 21.915) | 0.729 |
| Percutaneous coronary intervention | –1.285 (–19.367, 16.798) | 0.885 |
| rSO2 INVOS™ | –0.257 (–1.259, 0.766) | 0.590 |
| Pre–ECPR score | 26.218 (–18.551, 70.986) | 0.237 |
| **In-hospital characteristics** |  |  |
| Days on ECMO | 0.843 (–0.357, 2.044) | 0.160 |
| Days on ventilator | 0.169 (–0.387, 0.725) | 0.536 |
| Length of ICU stay, days | 0.144 (–0.364, 0.651) | 0.564 |
| Length of hospital stay, days | 0.020 (–0.248, 0.289) | 0.877 |
| In hospital rehabilitation | 0.200 (–18.171, 18.571) | 0.982 |
| Renal replacement therapy | –8.833 (–26.371, 8.705) | 0.309 |
| ICU delirium | 2.464 (–15.434, 20.363) | 0.779 |
| Neurological insult at ICU | 7.273 (–10.557, 25.102) | 0.408 |
| Heart transplantation | 13.579 (–5.742, 32.900) | 0.160 |
| Accidental hypothermia | –9.833 (–43.118, 23.451) | 0.548 |
| LVAD | 11.017 (–1.840, 23.874) | 0.090 |
| Cardiac rehabilitation | 8.575 (–9.437, 26.587) | 0.336 |
| Neurorehabilitation | 1.869 (–16.042, 19.780) | 0.831 |
| **Physical characteristics** |  |  |
| Follow up time, years | –0.468 (–2.980, 2.045) | 0.704 |
| Age at follow up | –0.228 (–0.997, 0.541) | 0.547 |
| Living at home | –2.120 (–25.341, 21.101) | 0.852 |
| Marital status | 2.073 (–15.997, 20.142) | 0.815 |
| Employed at follow up | –5.223 (–9.205, –1.242) | 0.012 |
| Return to work | 2.859 (–11.614, 17.333) | 0.687 |
| Moderate physical activity days/week | 6.036 (3.141, 8.931) | <0.001 |
| Vigorous physical activity days/week | 3.386 (–0.740, 7.512) | 0.103 |
| Six min walk test, meters | 0.040 (–0.028, 0.107) | 0.231 |
| 5 x Sit to stand, seconds | –0.894 (–1.746, –0.041) | 0.041 |
| Two simple questions – help needed | –27.217 (–52.735, –1.700) | 0.038 |
| Two simple questions – mentally recovered | 3.451 (–15.279, 22.181) | 0.707 |

No adjustment for multiple comparisons was made.

Abbreviations: CA, cardiac arrest; CI, confidence interval; CPR, cardiopulmonary resuscitation; ECMO, extracorporeal membrane oxygenation; ECPR, extracorporeal cardiopulmonary resuscitation; EQ-5D-5L, EuroQol 5-dimension 5-level; ICU, intensive care unit; LVAD, left ventricular assist device; OHCA, out-of-hospital cardiac arrest; rSO2, regional cerebral oxygen saturation; VAS, visual analogue scale.

|  |  |
| --- | --- |
| Physical function | 90 |
| Role physical | 100 |
| Bodily pain | 100 |
| General health | 65 |
| Vitality | 80 |
| Social functioning | 87 |
| Role emotional | 100 |
| Mental health | 84 |

**Figure S1.** 36 Item Short Form Health Survey (SF-36) scores. Spider plot of median SF-36 scores.
